# The Many Faces of Stress: Preliminary Validation of a Remote Photoplethysmography-Based Tool for Psychophysiological Stress and Emotional Distress Monitoring

**DOI:** 10.3390/healthcare14131893

**Published:** 2026-06-29

**Authors:** Livio Provenzi, Valeria Calcaterra, Sarah Nazzari, Paolo Osvaldo Agnelli, Marco Xodo, Sergio De Pasquale, Gianvincenzo Zuccotti

**Affiliations:** 1 Department of Brain and Behavioral Sciences, University of Pavia, 27100 Pavia, Italy; livio.provenzi@unipv.it; 2 Developmental Psychobiology Lab, IRCCS Mondino Foundation, 27100 Pavia, Italy; 3 Pediatric and Adolescent Unit, Department of Internal Medicine, University of Pavia, 27100 Pavia, Italy; valeria.calcaterra@unipv.it; 4 Pediatric Department, Buzzi Children’s Hospital, 20154 Milano, Italy; 5 Come Stai S.p.A, 20132 Milano, Italy; poa@comestai.io (P.O.A.); marco.xodo@comestai.io (M.X.); depa@lavoro.com (S.D.P.); 6 Department of Biomedical and Clinical Science, University of Milano, 20154 Milano, Italy

**Keywords:** stress, app, remote photoplethysmography, psychophysiological stress, exploratory validation, monitoring

## Abstract

**Highlights:**

**What are the main findings?**

- A mobile rPPG-based app provides feasible and user-friendly non-contact monitoring of psychophysiological stress in real-world settings.
- The Stress Level index is significantly associated with anxiety, depression, and general emotional distress, and is the only independent predictor in regression models.

**What are the implications of the main findings?**

- rPPG-based tools may provide preliminary complementary physiological information on stress-related autonomic activation beyond self-report measures, but they should not be interpreted as validated diagnostic or predictive tools.
- Longitudinal studies with repeated rPPG assessments and validation against reference physiological measures are required before these technologies can be considered for early detection or preventive mental health applications.

**Abstract:**

Background: Chronic stress contributes to mental and physical disorders, including burnout, anxiety, and depression. While self-report assessments remain valuable, they are inherently subjective and may be insensitive to short-term psychophysiological fluctuations. Remote photoplethysmography (rPPG) enables non-contact extraction of cardiovascular signals from facial videos and has increasingly been explored for stress-related monitoring through heart rate and heart rate variability features. Objective: This preliminary study aimed to assess the feasibility, usability, and preliminary construct validity of a mobile rPPG-based application for psychophysiological stress monitoring in daily life by examining usability, stress index distributions, and associations with self-reported psychological distress. Methods: A total of 252 participants from the general population and university students completed standardized facial video acquisition using a smartphone-based rPPG application and self-report questionnaires. The app extracted pulse wave signals, computed cardiovascular features related to heart rate and pulse rate variability, and integrated them into three indices: Stress Level, Stress Recovery, and Stress Response. Correlation and regression analyses examined associations with psychological distress. Results: The three indices showed substantial inter-individual variability. Stress Level was significantly associated with anxiety (r = 0.13, *p* = 0.036), depressive symptoms (r = 0.13, *p* = 0.047), and General Emotional Distress (r = 0.17, *p* = 0.006). In regression analysis, Stress Level emerged as the only significant independent correlate of General Emotional Distress (β = 0.21, *p* = 0.017). Younger participants and women showed higher Stress Level scores. Conclusions: The present findings should therefore be interpreted as preliminary and exploratory evidence of construct validity, suggesting that the app-derived indices may capture individual differences in stress-related physiological activation in everyday contexts. Currently, the observed associations were weak, the model explained limited variance, and the results do not demonstrate clinical validity, diagnostic utility, or predictive accuracy. Looking ahead, further longitudinal studies, repeated rPPG assessments, correction-aware analyses, and validation against reference physiological measures are needed before these indices can be considered suitable for clinical or preventive use.

## 1. Introduction

Mental health and stress regulation are central issues in both clinical and everyday contexts [1,2,3]. Mental disorders affect millions of individuals worldwide, and symptoms of stress, anxiety, and depression represent the most prevalent psychological conditions in the general population [4,5,6]. Certain groups appear particularly vulnerable, including university students, who are exposed to high academic demands, performance pressure, and uncertainty about future career prospects [7,8,9,10].

It is now widely recognized that prolonged, chronic, or excessive exposure to stress may have serious consequences for psychological well-being, representing a fertile ground for the development of emotional disorders and stress-related syndromes such as burnout [11,12]. From a psychophysiological perspective, sustained stress exposure has been linked to alterations in autonomic nervous system regulation, including changes in heart rate variability (HRV), cardiovascular reactivity, and stress-response system functioning [13,14,15,16]. These processes are considered relevant theoretical mechanisms, although they were not directly measured in the present study. For these reasons, cautious identification and monitoring of stress-related signals may be useful within broader multidimensional assessments, but such monitoring should not be equated with early diagnosis or prevention without prospective validation.

In this context, the possibility of integrating physiological correlates of stress with self-report measures becomes increasingly relevant. Traditional assessment of stress largely relies on self-report questionnaires, which, although valuable and clinically informative, are inherently limited by recall bias, subjectivity, and reduced sensitivity to short-term fluctuations [17,18]. Physiological indicators may therefore provide complementary information and contribute to a more comprehensive and ecologically valid evaluation of stress-related processes [19,20,21]. In particular, heart rate (HR) and HRV are well-established markers of autonomic regulation and stress-related physiological activation.

Remote photoplethysmography (rPPG) has recently emerged as a promising non-contact, low-cost, and scalable method to extract vital parameters such as HR, HRV, respiratory signals, and other hemodynamic indices directly from facial videos [22,23,24]. Previous studies have demonstrated the feasibility and accuracy of rPPG-derived cardiovascular measurements, particularly HR and HRV, under controlled and semi-ecological conditions. In addition, several recent studies have explored the use of rPPG-derived HR and HRV features combined with machine-learning approaches for stress detection and classification. However, rPPG performance may be affected by motion artifacts, illumination variability, facial positioning, and camera quality, and evidence regarding its application to mental-health monitoring is still emerging [22,23,24,25,26,27,28,29,30]. Thus, rPPG-based stress monitoring should currently be considered a promising complementary approach rather than a tool ready for widespread clinical implementation. Recent protocols have also shown the feasibility of integrating rPPG into mobile and digital health platforms for the collection of physiological and clinically relevant data in real-world and remote settings [21,22,23,24].

Although stress-related physiological activation can be inferred from cardiovascular features extracted through rPPG [31,32,33,34,35], these systems do not directly measure psychological stress or burnout and should therefore be interpreted within a broader multidimensional assessment framework. In this context, innovative technologies may help characterize stress-related physiological patterns; however, their use in preventive decision-making requires further longitudinal evidence and validation against reference criteria.

The aim of the present preliminary and exploratory study was therefore to assess the feasibility, usability, and preliminary construct validity of a mobile, consumer-oriented rPPG application in ecologically relevant daily life conditions. Specifically, the study aimed to evaluate user acceptability, describe the distribution of rPPG-derived stress-related physiological indices, and explore their cross-sectional associations with psychological, sociodemographic, and lifestyle variables. Furthermore, the study investigated the association between app-derived physiological indices and a composite General Emotional Distress score, conceptualized as an exploratory indicator of shared anxiety and depressive symptoms rather than a clinical measure of burnout risk or a predictive outcome.

## 2. Materials and Methods

### 2.1. Participants

A total of 252 participants took part in the study (range = 19–74 years), of whom 33.3% were male. Participants were consecutively recruited between March 2025 and May 2025 from two sources: (a) 100 individuals from the general population through the Norstat research panel; (b) 152 university students recruited at the University of Pavia, Italy.

Eligibility criteria included being aged 18 years or older, being able to understand the study procedures, providing written informed consent, and being able to complete both the self-report questionnaires and the app-based rPPG assessment. In line with the acquisition protocol previously described for this mobile rPPG-based system [24], participants also had to be able to perform the facial scan under appropriate recording conditions, including adequate face positioning, minimal movement, and sufficient facial visibility.

Participants were also excluded if they self-declared any known psychiatric disorder, cardiovascular disease, medication use, substance use, or other conditions that could substantially affect physiological stress assessment.

Thus, only participants with complete data for both the self-report measures and the app-based physiological assessment and without declared exclusion conditions were included in the analyses. Because exclusion criteria were based on self-report rather than clinical verification or medical records, residual misclassification bias cannot be excluded.

Lifestyle-related variables, including smoking status and alcohol or caffeine consumption, were collected for descriptive purposes but were not used as exclusion criteria unless participants reported substance use or clinically relevant conditions. Recruitment source was recorded, and differences between the two subsamples on the main study variables were inspected as detailed below. All participants received detailed information about the study procedures and provided written informed consent prior to participation. All data were collected and stored in anonymized form, and confidentiality was maintained in accordance with applicable data protection regulations. Given the use of smartphone-based facial physiological data, data privacy and algorithmic transparency were considered important ethical aspects. Participants were also informed about the non-diagnostic nature of the app-derived indices.

### 2.2. Procedure and Measures

#### 2.2.1. Self-Report Measures

Participants completed a battery of well-validated self-report questionnaires assessing psychological dimensions and lifestyle variables. Specifically, as part of the broader assessment, participants provided lifestyle-related data, including sleep, physical activity, smoking status, alcohol and caffeine consumption, BMI, and medication use. These variables were collected for descriptive and exploratory purposes, but were not included in the current analyses because they were outside the scope of the present manuscript.

For the purposes of the current work, we focused on measures of anxiety and depressive symptoms. Anxiety symptoms were assessed using the validated Italian version of the Generalized Anxiety Disorder 7-item scale (GAD-7) [36]. The GAD-7 consists of seven items rated from 0 (‘not at all’) to 3 (‘nearly every day’), yielding a total score ranging from 0 to 21, with higher scores indicating greater anxiety symptom severity. Depressive symptoms were assessed using the Italian version of the Beck Depression Inventory-II (BDI-II) [37]. The BDI-II consists of 21 items rated from 0 to 3, yielding a total score ranging from 0 to 63, with higher scores indicating greater depressive symptom severity. Internal consistency in the present sample was good for the GAD-7, Cronbach’s α = 0.86, and excellent for the BDI-II, Cronbach’s α = 0.91.

Given that no burnout-specific questionnaire was administered, anxiety and depressive symptoms were considered as indicators of general emotional distress rather than as direct measures of burnout. Accordingly, the derived emotional distress score was interpreted as a proxy indicator of burnout-related psychological vulnerability, reflecting the shared variance between anxiety and depressive symptomatology.

#### 2.2.2. Digital Assessment and Data Acquisition

The rPPG assessment was completed immediately before the self-report questionnaires, within the same assessment session. Thus, physiological recordings and questionnaire data were collected contemporaneously, with no relevant delay between the two procedures. Participants completed a single facial scan using a mobile application based on rPPG technology (r-PPG comestai_app V4.6, SDK 5.1.1.4), which extracts physiological parameters from facial video recordings acquired through the smartphone front-facing camera, without physical contact or wearable sensors. The application used in the present study was based on a system previously described and validated in independent studies [24].

Each facial recording lasted approximately 90 s (about 1.5 min), as required by the application protocol. The analytic dataset included one valid recording per participant. A total of 252 participants were enrolled, and 252 valid rPPG recordings were available for analysis; therefore, no recordings were excluded because of inadequate signal quality (0/252; 0.0%). Repeated attempts were permitted only when the application did not complete the acquisition or did not return a valid output because the scan failed the automated quality-control checks; in such cases, participants were instructed to repeat the scan after correcting face position, lighting, or movement. No failed or incomplete scans were retained for analysis.

Before recording, participants followed standardized in-app instructions. They were asked to sit in a quiet indoor environment, remain still, avoid speaking, and minimize facial or head movements. They were instructed to position their face within the on-screen frame, keep the smartphone stable at an appropriate distance from the front-facing camera, and look directly at the screen throughout the recording. Adequate and stable lighting was required, avoiding direct sunlight, strong backlighting, facial shadows, or abrupt changes in illumination, while keeping the forehead and cheeks visible to support facial region detection and rPPG signal extraction. A single facial scan was performed for each participant under real-world but standardized conditions [24]. However, illumination was not quantified using a lux meter or experimentally standardized, which should be considered a methodological limitation.

The predefined criteria for an acceptable acquisition were: full facial visibility, stable face detection within the on-screen frame, adequate visibility of forehead and cheek regions, minimal head or facial movement, absence of speaking during the scan, stable smartphone positioning, and sufficiently homogeneous illumination without strong shadows, direct sunlight, or backlighting. Recordings that did not satisfy these requirements were automatically flagged by the application and were not saved as valid scans.

Participants completed the rPPG assessment using their own smartphones through the mobile application, in an unsupervised, app-supported, real-world setting, with acquisition procedures and quality-control steps described below. Thus, device type was not standardized across participants, and both Android and iOS smartphones were used, reflecting conditions closer to real-world use. Phone make/model and operating system were not experimentally controlled. Consequently, technical characteristics such as camera model, resolution, frame rate, exposure settings, and brightness regulation may have varied across devices. Recordings were expected to be obtained at approximately 30 frames per second, consistent with typical smartphone front-facing camera acquisition, although the exact frame rate and resolution could differ depending on the device and operating system. This approach increases ecological validity; however, device heterogeneity should be acknowledged as a methodological limitation, as differences in camera hardware and acquisition parameters may affect rPPG signal quality.

#### 2.2.3. Signal Processing and Stress Index Computation

The signal processing and stress index computation pipeline implemented in the application follows the general and well-established principles of rPPG. Facial videos acquired through the smartphone front-facing camera are first processed through an automated preprocessing and quality-control stage. This stage includes face detection, verification of facial positioning within the camera frame, identification of suitable skin regions, and selection of informative regions of interest (ROIs), primarily located on facial areas with favorable optical properties for rPPG signal extraction, such as the forehead and cheeks. Continuous ROI tracking and stabilization are applied across consecutive frames to preserve spatial consistency and reduce the influence of small head movements, facial displacement, and transient changes in alignment.

Quality control was performed before index computation. The application verified face presence and stability, ROI tracking continuity, lighting stability, motion artifact burden, waveform plausibility, and algorithmic confidence. A recording was considered acceptable only if it passed the internal signal-quality check and generated valid Stress Level, Stress Recovery, and Stress Response outputs. The numerical cut-off values used by the software for the signal-quality score, motion rejection, ROI stability, and algorithmic confidence are proprietary and cannot be disclosed; however, the pass/fail decision was applied automatically and identically to all participants before data export.

In the subsequent stage, temporal color traces are extracted from the selected ROIs by analyzing frame-by-frame variations in the RGB channels. These subtle color fluctuations reflect changes in light absorption associated with pulsatile blood volume variations in the superficial facial microvasculature. The resulting raw signals are then subjected to normalization and temporal filtering procedures aimed at enhancing the cardiac-related pulsatile component while attenuating non-physiological noise sources, including motion artifacts, illumination changes, shadowing effects, and camera sensor variability. Additional artifact-reduction procedures are applied to improve waveform stability and to exclude signal segments affected by poor tracking, excessive movement, or insufficient optical quality.

After preprocessing, the cleaned rPPG waveform is analyzed to extract physiologically relevant cardiovascular features. These may include HR, pulse waveform characteristics, and time- and/or frequency-domain indices related to beat-to-beat variability. Such features provide an indirect representation of autonomic cardiovascular regulation and are used as the physiological basis for the computation of higher-level synthetic indices.

The stress index is then estimated through proprietary computational models that integrate the extracted rPPG-derived cardiovascular features into composite stress-related indices. These indices are intended to capture different aspects of autonomic and cardiovascular regulation associated with stress-related physiological states [21]. In this framework, temporal and morphological characteristics of the cleaned waveform are combined with measures of beat-to-beat variability, signal stability, ROI consistency, and algorithmic confidence to generate a synthetic estimate of stress-related physiological activation. Although the general principles of rPPG technology and the acquisition protocol have been described in previous publications, the specific details of the signal-processing pipeline, feature weighting, and computational models used to derive the final indices are part of a commercially protected system and therefore cannot be fully disclosed. Nevertheless, the system is grounded in established rPPG methodologies and has previously been evaluated in terms of accuracy and feasibility against standard reference devices [21,24,38].

For participant-flow reporting, recordings that failed acquisition or quality control would have been classified as poor-quality scans and excluded from the analytic dataset unless a successful repeat scan was obtained. In the present sample, all included participants provided one valid recording, and the number of exclusions due to inadequate rPPG signal quality was zero.

The Comestai application received CE Medical Device Regulation (MDR) Class 1 certification in Italy, identified by the registration number R/2741800 in the Italian Ministry of Health.

#### 2.2.4. Conceptual Definition of the App-Derived Stress Indices

The application provides three main physiological stress-related indices derived from the processing of facial video recordings. Stress Level is defined as a concurrent estimate of stress-related autonomic activation, primarily reflecting patterns such as higher HR, lower beat-to-beat variability, and reduced cardiovascular flexibility. Stress Recovery reflects recovery-related autonomic regulation, with higher scores indicating more favorable recovery capacity and lower scores suggesting reduced recovery-related functioning. Stress Response represents physiological reactivity, intended to capture the magnitude of the autonomic cardiovascular response to potentially stressful conditions. These definitions are conceptual rather than independently validated computational decompositions, because the exact feature weighting and transformations remain proprietary.

All app-derived indices are expressed on a standardized 0–100 scale. Higher scores on Stress Level and Stress Response correspond to greater physiological stress activation and stronger reactivity, respectively. By contrast, higher Stress Recovery scores indicate more favorable recovery-related functioning, whereas lower scores suggest reduced recovery capacity. In the present study, the indices were analyzed in their original app-generated form and were not additionally adjusted for age, sex, recruitment source, or other individual-level covariates.

The Stress Level index is estimated through computational models that integrate rPPG-derived cardiovascular features into a composite stress-related indicator [21]. After extraction and cleaning of the pulse waveform, the system identifies consecutive pulse peaks and derives beat-to-beat pulse intervals, which are used as rPPG-based surrogates of RR intervals, namely the time intervals between consecutive heartbeats. From these interval series, the algorithm estimates HR and HRV parameters reflecting short-term autonomic cardiovascular regulation. These parameters may include time-domain indices such as the standard deviation of normal-to-normal intervals (SDNN), reflecting overall variability, and the root mean square of successive differences (RMSSD), reflecting short-term beat-to-beat variability, as well as frequency-domain components related to sympatho-vagal balance, such as the low-frequency/high-frequency ratio (LF/HF). In addition, the temporal distribution of RR/pulse intervals, including measures of interval dispersion, concentration around the modal value, and variability range, can be used to characterize the regularity or flexibility of cardiac rhythm.

The physiological rationale underlying this approach is that higher stress-related autonomic activation is generally associated with increased HR, reduced HRV, a more regular and less variable interval distribution, and a shift toward sympathetic predominance with reduced parasympathetic modulation. Accordingly, the Stress Level index combines HR, HRV, and RR/pulse interval-derived features into a synthetic estimate of stress-related autonomic activation. Although the exact feature weighting and computational implementation remain proprietary, the index is grounded in established HRV-based principles of physiological stress assessment and is intended to summarize cardiovascular variability patterns associated with psychophysiological stress responses.

#### 2.2.5. Evaluation of Usability and User Experience

Usability was evaluated using a 5-item ad hoc questionnaire rated on a 7-point Likert scale (1 = strongly disagree; 7 = strongly agree) [39]. The items were designed de novo for the purposes of the present study and were not adapted from an existing standardized usability instrument. The questionnaire assessed participants’ perceived ease of use of the tool, clarity of the information provided, perceived safety and reliability of the system, the overall quality of the scanning experience, and the intention to reuse the application in the future. An overall usability score was computed by averaging the five items, with higher scores indicating a more positive usability evaluation. Usability results were examined descriptively and were not included in the main inferential analyses. Internal consistency of the scale was borderline for preliminary research purposes, Cronbach’s α = 0.67; therefore, usability findings should be interpreted strictly as exploratory user-experience observations rather than as validated usability evidence.

### 2.3. Statistical Analyses

All statistical analyses were conducted using standard statistical software packages. First, descriptive statistics (means, standard deviations, frequencies, and percentages) were computed to characterize the sample in terms of sociodemographic variables, psychological measures, and app stress indices. Analyses were conducted using a complete-case approach, including only participants with complete self-report and app-derived rPPG data.

Before conducting inferential analyses, the distribution of the main continuous variables was inspected visually using histograms and Q–Q plots and formally evaluated using the Shapiro–Wilk test. Homogeneity of variance was assessed before analysis of variance procedures. For regression analyses, residual diagnostics were inspected to evaluate linearity, normality of residuals, homoscedasticity, and the presence of influential observations. When assumptions were not fully met, findings were interpreted cautiously, and non-parametric alternatives were considered as sensitivity checks.

Multicollinearity among predictors was assessed by inspecting variance inflation factor (VIF) and tolerance values. VIF values below 5 and tolerance values above 0.20 were considered to indicate the absence of problematic multicollinearity.

The distribution of the three App indices (Stress Level, Stress Recovery, and Stress Response) was examined using frequency distributions and descriptive indicators in order to assess inter-individual variability and identify potential skewness or ceiling/floor effects.

Group differences in stress indices as a function of sociodemographic variables were tested using analysis of variance (ANOVA) for continuous variables (e.g., age groups) and chi-square (χ^2^) tests for categorical variables (e.g., gender). When appropriate, post hoc comparisons were performed to further explore significant effects.

Associations between app-derived stress indices and self-report psychological measures, namely anxiety and depressive symptoms, were examined using Pearson’s correlation coefficients when assumptions of approximate normality and linearity were considered acceptable. When these assumptions were not adequately met, non-parametric Spearman correlations were considered. Effect sizes were interpreted according to conventional criteria.

To derive a synthetic indicator of general emotional distress, anxiety and depressive symptom scores were standardized and averaged to obtain a composite General Emotional Distress score. Because this score was based on only two closely related psychological variables, it was not interpreted as a distinct latent construct. The composite was used in subsequent analyses as an exploratory indicator of shared anxiety-depression burden and as a proxy indicator of burnout-related psychological vulnerability, rather than as a direct measure of burnout.

Finally, a multiple linear regression model was estimated to test the predictive value of the three App stress indices (Stress Level, Stress Recovery, Stress Response) on General Emotional Distress [40]. Regression coefficients, standard errors, 95% confidence intervals, standardized beta coefficients, and *p*-values were reported. Overall model fit was evaluated using the F-statistic and corresponding *p*-value.

As a sensitivity analysis, the regression model was repeated by including recruitment source as an additional covariate in order to examine whether the associations between app-derived stress indices and General Emotional Distress remained stable after controlling for potential recruitment-source confounding.

Given the exploratory nature of the study and the number of correlations, group comparisons, and regression analyses conducted, the risk of type I error due to multiple testing was acknowledged. No formal correction for multiple comparisons was applied; therefore, statistically significant findings were interpreted cautiously and should be considered exploratory, hypothesis-generating, and in need of replication in adequately powered confirmatory studies.

All tests were two-tailed, and the level of statistical significance was set at *p* < 0.05.

## 3. Results

### 3.1. Study Population

A total of 252 participants took part in the study (mean age 30.8 years, SD = 14.0), of whom 33.3% were male. The sample was heterogeneous in terms of age, gender, and sociodemographic background and included students, workers, and unemployed individuals. With respect to educational attainment, 68.3% of the sample had completed upper secondary education, 27.4% held at least a university degree, whereas only a small minority had only completed middle school (4.0%) or primary school education (0.4%).

Regarding occupational status, the sample consisted mainly of students (57%), followed by workers (31%), and a smaller proportion of unemployed individuals (5%), confirming the predominance of young adults and individuals in training or early career stages within the study population.

### 3.2. Usability of the App

Overall, the app was perceived as easy to use, intuitive, and understandable. Specifically, 74% of participants reported that the app was easy and intuitive from the first use (mean score, M = 5.18; SD = 1.44), and 83% reported that the information provided was clear and comprehensible (M = 5.40; SD = 1.18). About 70% of participants judged the scanning procedure as fast and not frustrating (M = 5.00; SD = 1.35). Perceived safety and reliability received moderate scores (M = 3.77; SD = 1.52), with most participants choosing central values of the scale, suggesting room for improvement in transparency and feedback communication. The intention to reuse the app was also moderate (M = 4.16; SD = 1.58), with a positive trend among older users, who appeared more confident and willing to integrate the app into their daily health monitoring routine.

### 3.3. Distribution of Stress Indices

**Figure 1:**
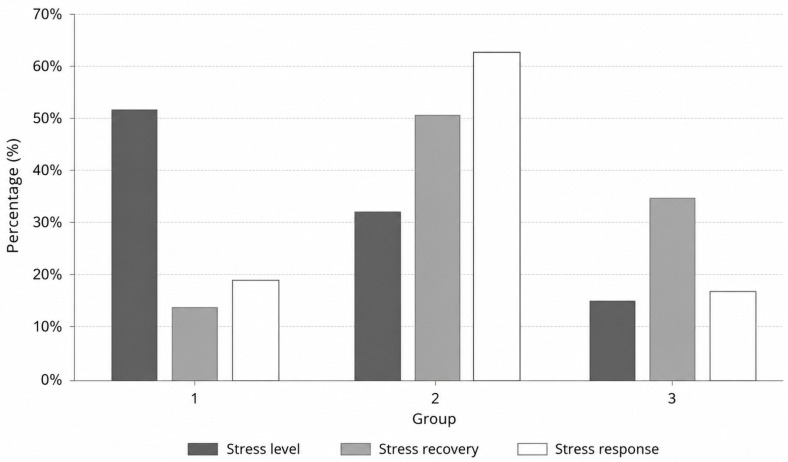
Percentage distribution of stress-related indices. The chart shows the percentage distribution of the three stress-related indices provided by the app, Stress Level, Stress Recovery, and Stress Response, across three descriptive groups—1 = low, 2 = medium, and 3 = high—highlighting the different percentage frequencies for each indicator. For Stress Level and Stress Response, higher group values indicate greater stress burden and stronger physiological reactivity, respectively. For Stress Recovery, higher group values indicate better recovery capacity, whereas lower values reflect poorer recovery capacity.

The three stress indices provided by the app (Stress Level, Stress Recovery, and Stress Response) showed substantial inter-individual variability, confirming the heterogeneity of psychophysiological stress profiles in the sample (Figure 1).

With respect to Stress Level, approximately 50% of participants fell within the medium-to-high range of the scale. Stress Recovery exhibited a markedly heterogeneous distribution, with approximately 35–40% of participants showing low recovery capacity. Stress Response scores spanned a wide range, with approximately 60% of participants located in the medium-to-high reactivity range, reflecting pronounced inter-individual differences in physiological stress responsivity.

Overall, the joint inspection of the three distributions indicates that the indices provide complementary information on stress exposure, reactivity, and regulatory capacity.

### 3.4. Stress Indices and Sociodemographic Variables

Significant sociodemographic effects emerged for Stress Level as a function of both age and gender. Younger participants reported significantly higher Stress Level scores compared to older participants (F = 5.81, *p* = 0.004), and female participants exhibited higher Stress Level scores than males (χ^2^ = 6.75, *p* = 0.034). A chi-square test of independence was conducted to examine the association between stress level and recruitment source. The association was statistically significant (χ^2^ = 17.10, *p* < 0.001), indicating that the distribution of stress levels differed significantly between the two subsamples. Inspection of the observed frequencies showed that participants from the student sample were more frequently classified in the highest stress category than participants from the panel sample. Specifically, 21.7% of participants in the student sample were classified as high stress, compared with 6.0% in the panel sample. Conversely, the panel sample showed a higher proportion of participants in the intermediate stress category than the student sample. No significant associations were found for Stress Response and Stress Recovery indices.

### 3.5. Stress Indices and Psychological Symptoms

**Figure 2:**
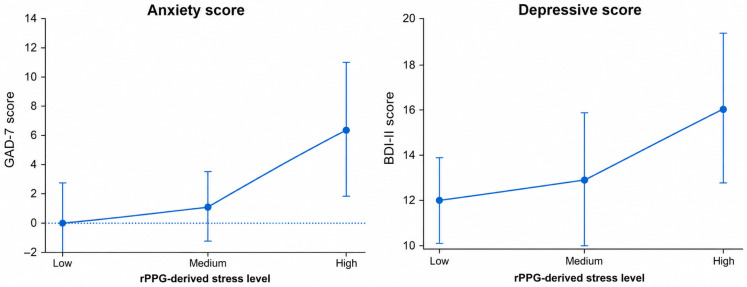
Mean psychological symptom scores across remote photoplethysmography (rPPG)-derived stress-level categories. Mean anxiety symptom scores, assessed using the Generalized Anxiety Disorder-7 scale (GAD-7), and mean depressive symptom scores, assessed using the Beck Depression Inventory-II (BDI-II), are displayed across low, medium, and high stress-level categories derived from rPPG. Points represent group mean values, while vertical bars indicate the 95% confidence intervals. The figure shows an increasing trend in both anxiety and depressive symptom scores across higher rPPG-derived stress levels. However, the overlap between confidence intervals indicates that these differences should be interpreted cautiously.

As illustrated in Figure 2, Stress Level was positively and significantly correlated with both anxiety symptoms (r = 0.13, *p* = 0.036) and depressive symptoms (r = 0.13, *p* = 0.047). No significant correlations were observed between anxiety or depressive symptoms and either Stress Recovery or Stress Response (all *p* > 0.10). In addition, we examined whether the two recruitment sources differed in anxiety symptoms, depressive symptoms, and General Emotional Distress. The distribution of anxiety and depressive symptoms did not significantly differ between the two subsamples (ps > 0.22). Likewise, no significant difference between the two subsamples was observed for the General Emotional Distress factor (*p* > 0.62).

### 3.6. Stress Indices and General Emotional Distress

**Figure 3:**
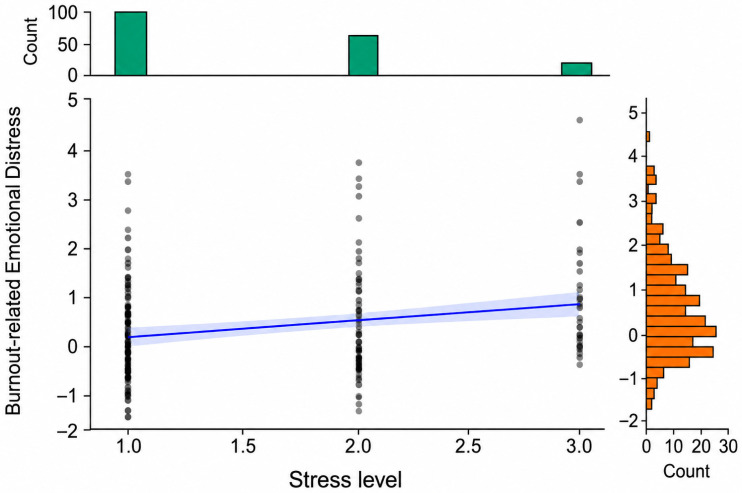
Association between stress level and General Emotional Distress. Each dot represents an individual observation. The blue line indicates the fitted linear trend, with the shaded area showing the 95% confidence interval. Marginal histograms display the distributions of stress levels (**top**) and emotional distress (**right**).

To investigate the relationship between the physiological stress indices and a broader dimension of general emotional distress, a composite General Emotional Distress score was examined. As shown in Figure 3, General Emotional Distress was significantly and positively correlated with Stress Level (r = 0.17, *p* = 0.006), while Stress Response showed a trend toward statistical significance (r = 0.12, *p* = 0.06); Stress Recovery was not significantly associated. These correlations were weak in magnitude and should be interpreted as exploratory.

### 3.7. Prediction of General Emotional Distress

A multiple linear regression model including the three stress indices was estimated on the full sample to examine their joint association with General Emotional Distress = β_0_ + β_1_(Stress Level) + β_2_(Stress Recovery) + β_3_(Stress Response) + ε [40].

The overall model was statistically significant, F(3, 248) = 3.28, *p* = 0.02, explaining approximately 4% of the variance in General Emotional Distress (R^2^ = 0.04). Importantly, only Stress Level emerged as a significant independent predictor (β = 0.21, *p* = 0.017; 95% CI = [0.05, 0.53]), whereas Stress Recovery (β = 0.13, *p* = 0.147) and Stress Response (β = 0.07, *p* = 0.467) did not reach statistical significance.

The pattern of results remained unchanged after including recruitment source as an additional covariate. The overall model was statistically significant, F(4, 248) = 2.77, *p* = 0.03, and only Stress Level emerged as a significant independent predictor (β = 0.23, *p* = 0.011; 95% CI = [0.07, 0.56]), whereas Stress Recovery (β = 0.15, *p* = 0.103) and Stress Response (β = 0.07, *p* = 0.469) remained non-significant.

Regression assumptions were further examined by assessing multicollinearity among the predictors. Collinearity diagnostics indicated no evidence of problematic multicollinearity. Variance Inflation Factor values were 2.03 for Stress Level, 2.10 for Stress Recovery, and 2.17 for Stress Response. Tolerance values were 0.494, 0.476, and 0.461, respectively. These values were within acceptable ranges, indicating that multicollinearity was unlikely to affect the stability of the regression estimates.

## 4. Discussion

The present study provides preliminary evidence supporting the feasibility, acceptability, and exploratory construct validity of the r-PPG comestai_app as a digital tool for psychophysiological stress-related monitoring in both general and student populations. These findings should not be interpreted as evidence of diagnostic validity, objective stress assessment, early detection, or predictive validity. Overall, the app was well received by users and demonstrated preliminary user acceptability, a prerequisite for digital health technologies intended for repeated use in everyday life contexts.

Beyond usability, the main empirical finding concerns the association between the Stress Level index and self-reported symptoms of anxiety, depression, and the composite indicator of General Emotional Distress. These associations were statistically significant but weak in magnitude. Thus, the convergence between app-derived physiological indices and psychometric measures should be interpreted as preliminary evidence that the app may capture limited variance in stress-related emotional distress, rather than as evidence of validated objective stress monitoring or clinically meaningful prediction [41].

From a psychobiological perspective, the observed associations can be discussed in relation to theoretical models linking stress-related physiological activation to emotional distress [42,43,44,45]. However, constructs such as HPA-axis dysregulation, allostatic load, inflammatory activation, and cumulative psychophysiological burden were not directly measured in the present study. Therefore, these mechanisms should be regarded as background explanatory frameworks only. The present findings do not demonstrate that the app directly measures chronic stress burden, allostatic load, cumulative dysregulation, or stress-system pathology. Moreover, no standard biological stress markers, such as cortisol or inflammatory markers, nor reference physiological measures, such as ECG-derived HRV or electrodermal activity, were collected in this study. Therefore, the Stress Level index should be interpreted as a preliminary app-derived physiological correlate potentially associated with stress-related autonomic activation, rather than as a proxy marker of cumulative psychophysiological burden [31,46,47].

Stress Level emerged as the only significant correlate of General Emotional Distress in the multivariate regression model, whereas Stress Recovery and Stress Response did not reach statistical significance. This finding supports the relevance of Stress Level as the app-derived measure most closely associated with concurrent emotional distress in the present sample, but it should be interpreted with caution because the model explained only 4% of the variance. The three indices are conceptually related and may share overlapping variance, which could have reduced the independent contribution of Stress Recovery and Stress Response in the multivariate model. Importantly, given the cross-sectional design, these results provide preliminary evidence of concurrent association, but do not establish predictive validity or causal relationships between app-derived indices and future burnout or mental health outcomes [48].

Burnout is increasingly conceptualized as a multifactorial syndrome resulting from prolonged exposure to unmanaged stress, particularly in academic and occupational settings characterized by high demands and limited resources [49,50,51]. Epidemiological studies have documented high rates of comorbidity between burnout, anxiety, and depression, as well as associations with cardiovascular, musculoskeletal, and metabolic disorders, and even increased risk of premature mortality [52,53,54]. In this context, the present findings suggest that r-PPG comestai_app may provide physiological information associated with concurrent emotional distress dimensions, although further evidence is needed before considering these indices as markers of burnout risk or long-term psychological vulnerability [31,47,55,56,57].

From an applied perspective, the results suggest potential but still unverified uses of brief rPPG-based assessments as complementary sources of physiological information [56,58]. Stress is not measured directly by PPG or rPPG; rather, stress-related autonomic activation may be indirectly inferred from HR and HRV features extracted from pulse wave signals and processed through computational models [21]. These physiological signals are not specific to psychological stress and may reflect multiple overlapping psychophysiological states. Therefore, rPPG-based indices should be interpreted within a broader multidimensional assessment framework integrating subjective, behavioral, clinical, and contextual information.

By providing app-derived physiological indicators in real-world settings, rPPG-based systems may complement traditional self-report assessments and support repeated ecological monitoring over time. In practical terms, such tools may help characterize stress-related physiological patterns, including sustained elevations or reduced recovery capacity, and could potentially contribute to supportive strategies such as stress-management prompts, breathing exercises, micro-breaks, or referral to support services [59]. However, because the present study was cross-sectional and based on a single spot assessment, it does not establish whether the app can identify early vulnerability trajectories, predict future mental health outcomes, guide preventive interventions, or capture temporal changes in stress accumulation and recovery. These potential applications therefore remain promising but hypothetical and require longitudinal, repeated-measure, and interventional validation before clinical implementation can be considered [60].

A potential value of the rPPG app approach lies in the integration of app-derived physiological indicators with subjective self-report measures [21,56]. This multi-level assessment framework is increasingly discussed in psychosomatic medicine, behavioral health, and precision psychiatry, as it may allow for a more comprehensive and individualized understanding of stress processes. In the present study, however, the convergence between app-based indices and psychological symptoms was weak; therefore, it supports only preliminary ecological plausibility, not established clinical validity [32,33,34,35,61].

It is also noteworthy that significant differences in Stress Level were observed as a function of age and gender, with younger individuals and women showing higher stress levels. This finding is consistent with the composition of the sample and with a large body of literature indicating that young adults, particularly university students, represent a high-risk group for stress-related psychological difficulties [62,63,64]. Because age, gender, recruitment source, and student status were partly intertwined in the present sample, these differences should be regarded as exploratory. Future studies with balanced and stratified samples are needed to determine whether age and gender independently contribute to rPPG-derived stress indices.

## 5. Limitations and Future Directions

Several limitations must be acknowledged. Overall, the present findings should be interpreted as preliminary evidence supporting the feasibility and exploratory construct validity of a smartphone-based rPPG approach for psychophysiological stress-related monitoring, rather than as evidence of clinical diagnostic validity, objective stress assessment, early detection, longitudinal risk prediction, or population-level prevention.

The cross-sectional design does not allow causal conclusions. The associations observed between app-derived stress indices and self-reported psychological distress should therefore be interpreted as concurrent relationships. Longitudinal studies are needed to determine whether rPPG-derived indices can predict changes in psychological distress or burnout-related outcomes over time.

The physiological assessment was based on a single rPPG recording for each participant. Consequently, test–retest reliability, within-subject stability, and day-to-day variability of the app-derived indices could not be evaluated. Future studies should include repeated assessments across different time points and contexts to examine the temporal stability of these measures.

Participants completed the assessment using their own smartphones. This approach increases ecological validity and reflects real-world use, but device type, camera hardware, frame rate, resolution, exposure settings, and operating system were not experimentally standardized. Environmental conditions were also not objectively quantified, despite participants being instructed to complete the scan in a quiet indoor environment with adequate and homogeneous lighting. In particular, illumination was not measured using a lux meter, and background noise or movement in the surrounding environment was not formally recorded. Skin tone and related optical-demographic characteristics were not collected, preventing evaluation of possible skin tone-related variability, differential signal quality, or demographic bias in rPPG signal extraction.

Quality-control procedures were applied before index computation, but they relied on automated internal checks implemented by the application. Recordings were considered acceptable only when face detection, facial positioning, ROI visibility and tracking, lighting stability, motion-artifact burden, waveform plausibility, and algorithmic confidence met the app-defined quality-control requirements. In the present sample, no recordings were excluded because of inadequate signal quality. However, the numerical thresholds used for signal-quality scoring, motion rejection, ROI stability, and algorithmic confidence are proprietary and could not be disclosed or externally evaluated, limiting full reproducibility of the quality-control decision rules.

The stress indices analyzed in the present study were generated through proprietary computational models. Although the general principles of rPPG signal acquisition and processing were described, the exact feature weighting, transformation procedures, algorithmic thresholds, and rejection rules underlying the final Stress Level, Stress Recovery, and Stress Response scores could not be independently verified. At this stage, these rPPG-derived indices should therefore be regarded as physiological correlates associated with concurrent self-reported distress, rather than as validated clinical markers or standalone predictors of burnout-related outcomes.

Exclusion criteria were based on self-reported known clinical conditions rather than on structured clinical interviews or medical record verification; therefore, residual misclassification bias cannot be excluded.

The psychological outcome used in the regression analyses was based on anxiety and depressive symptoms and was interpreted as a composite General Emotional Distress score. Although this composite may represent a proxy indicator of burnout-related psychological vulnerability, it should not be considered a direct measure of burnout, as no burnout-specific questionnaire was administered. Moreover, the GAD-7 and BDI-II assess anxiety and depressive symptoms, respectively, and should not be interpreted as stress-specific measures. The absence of a stress-specific questionnaire designed for the general population, such as the Perceived Stress Scale, DASS-21, or K-10, further limits the interpretation of the findings, which cannot be taken as evidence of an association between rPPG-derived indices and directly measured perceived stress. Recruitment source was examined in sensitivity analyses, and the pattern of results remained unchanged after including it as a covariate; nevertheless, residual confounding cannot be excluded. Further validation studies, including standardized stress and burnout measures, reference physiological assessments, preregistered analytic plans, correction for multiple testing, and models adjusted for recruitment source and other relevant covariates, are needed to determine the stability, reproducibility, fairness, and clinical relevance of these app-derived measures.

## 6. Conclusions

The rPPG-based comestai_app may represent a useful exploratory tool for assessing psychophysiological stress and General Emotional Distress. Its accessibility and smartphone-based format make it potentially relevant for digital mental health research. However, its practical role in preventive or clinical contexts remains to be established.

The present findings should therefore be interpreted with caution. They are based on cross-sectional, single-time-point assessments and do not demonstrate continuous monitoring capabilities, early identification of vulnerability trajectories, or prediction of future burnout-related outcomes. Moreover, the use of proprietary algorithms and the absence of longitudinal validation limit the extent to which these app-derived indices can currently inform prevention, screening, or long-term monitoring.

Further longitudinal studies, repeated rPPG assessments, transparent reporting of acquisition and quality-control procedures, and validation against reference physiological measures are needed to determine the stability, reproducibility, fairness, and clinical relevance of these indices before considering their use in preventive mental health care or long-term outcome monitoring.

## Data Availability

The datasets generated and analyzed during the current study are available from the corresponding author upon reasonable request due to ethical reasons. All data have been anonymized to protect participant privacy and comply with applicable ethical standards.
